# Towards personalized medicine in Ménière’s disease

**DOI:** 10.12688/f1000research.14417.1

**Published:** 2018-08-15

**Authors:** Jose Antonio Lopez-Escamez, Angel Batuecas-Caletrio, Alexandre Bisdorff

**Affiliations:** 1 Otology & Neurotology Group CTS495, Department of Genomic Medicine, Centro de Genómica e Investigación Oncológica, Pfizer/Universidad de Granada/Junta de Andalucía (GENYO), Granada, Spain; 2 Luxembourg Centre for Systems Biomedicine (LCSB), University of Luxembourg, Esch-sur-Alzette, Luxembourg; 3 Department of Otolaryngology, Instituto de Investigación Biosanitaria, ibs.GRANADA, Hospital Universitario Virgen de las Nieves, Universidad de Granada, Granada, Spain; 4 Department of Otolaryngology, Hospital Universitario de Salamanca, IBSAL, Salamanca, Spain; 5 Clinique du Vertige, Centre Hospitalier Emile Mayrisch, Esch-sur-Alzette, Luxembourg

**Keywords:** precision medicine, Meniere disease, vertigo, tinnitus, sensorineural hearing loss, molecular genetics, genomics

## Abstract

Ménière’s disease (MD) represents a heterogeneous group of relatively rare disorders with three core symptoms: episodic vertigo, tinnitus, and sensorineural hearing loss involving 125 to 2,000 Hz frequencies. The majority of cases are considered sporadic, although familial aggregation has been recognized in European and Korean populations, and the search for familial MD genes has been elusive until the last few years. Detailed phenotyping and cluster analyses have found several clinical predictors for different subgroups of patients, which may indicate different mechanisms, including genetic and immune factors. The genes associated with familial MD are *COCH*, *FAM136A*, *DTNA*, *PRKCB*, *SEMA3D*, and *DPT*. At least two mechanisms have been involved in MD: (a) a pro-inflammatory immune response mediated by interleukin-1 beta (IL-1β), tumor necrosis factor alpha (TNFα), and IL-6, and (b) a nuclear factor-kappa B (NF-κB)-mediated inflammation in the carriers of the single-nucleotide variant rs4947296. It is conceivable that microbial antigens trigger inflammation with release of pro-inflammatory cytokines at different sites within the cochlea, such as the endolymphatic sac, the stria vascularis, or the spiral ligament, leading to fluid imbalance with an accumulation of endolymph. Computational integration of clinical and “omics” data eventually should transform the management of MD from “one pill fits all” to precise patient stratification and a personalized approach. This article lays out a proposal for an algorithm for the genetic diagnosis of MD. This approach will facilitate the identification of new molecular targets for individualized treatment, including immunosuppressant and gene therapy, in the near future.

## Introduction

Computational biomedicine integrating clinical and “omics” data is leading to molecular, individualized patient-oriented treatments, replacing the traditional approach based on clinical symptoms and few laboratory or imaging markers. Ménière’s disease (MD) is a set of rare inner ear disorders defined by a core phenotype: (a) episodes of vertigo associated with ipsilateral cochlear symptoms, such as tinnitus or aural fullness, and (b) sensorineural hearing loss (SNHL), which initially fluctuates and involves low and medium frequencies ^1, 2^. However, the condition exhibits a great clinical heterogeneity and large differences in response to therapy. The disorder is commonly explained by the accumulation of endolymph with increased pressure in the cochlear duct (endolymphatic hydrops [EH]), which damages the organ of Corti, the hearing organ; this overpressure leads to the rupture of the inner ear membranes, resulting in a loss of endocochlear potential in humans. The damage involves the scala media of the cochlea and the vestibular end organs (saccule and utricle) and eventually the semicircular canals ^3^. However, the clinical heterogeneity of MD is born in the pathophysiology itself and EH cannot explain the episodes of vertigo. Some patients with demonstrated EH do not develop all MD symptoms, whereas in some patients with definite MD an EH cannot be demonstrated ^4^. For this reason, EH is considered to be a marker of some underlying pathological process that is related to MD, such as a disorder of endolymphatic fluid homeostasis ^5^.

Repeated exposure of hair cells to toxic levels of a K ^+^-enriched perilymph, the overpressure itself, and the sudden rupture of distended membranes explain the long-term vestibular and auditory damage in MD ^6, 7^.

The treatment of MD is based on empirical clinical practice. There is some evidence of therapies reducing the number of attacks, including trans-tympanic steroids ^8^, but so far none has been able to prevent progressive damage to the inner ear.

The prevalence of MD is variable, ranging from 3.5 per 100,000 inhabitants in Japan to 513 per 100,000 in Finland ^9– 15^. The condition shows differences in its prevalence according to ethnic background, it is more commonly observed in European than in Asian or American populations ^16, 17^, and it is rarely found in sub-Saharan populations, suggesting an ethnic-mediated genetic contribution. These differences in prevalence could also be explained by differences in availability of health care, but the finding of familial aggregation and multiple families in European and Korean populations supports the genetic background in MD ^18, 19^.

Furthermore, deep phenotyping and cluster analyses have found few clinical predictors for several subgroups of patients with MD, which may indicate different mechanisms of disease, including genetic and autoimmune factors ^20, 21^.

We describe the evidence supporting the possibility that MD is a heterogeneous disorder with several clinical variants, such as familial, autoimmune, and auto-inflammatory MD. We show the preliminary data that support the genetic contribution to MD and present a tentative algorithm for the genetic diagnosis of MD. This approach will facilitate the identification of new molecular targets for an individualized treatment.

## Diagnosis of Ménière’s disease

The diagnostic criteria for MD were revised in 2015 by a Joint Consensus agreed on by five international scientific societies: the Barany Society, the Korean Balance Society, the Japan Society for Equilibrium Research, the European Academy of Otology and Neurotology, and the Equilibrium Committee of the American Academy of Otolaryngology-Head and Neck Surgery ^1^. Two diagnostic categories were accepted: definite MD and probable MD ( Table 1). These criteria are based on clinical symptoms and do not consider different subgroups of patients, laboratory markers (except pure tone audiograms), or imaging data. Although the aim of the revised diagnostic criteria is to gain precision in the diagnosis by improving the phenotyping, no “omics” data were available in 2015 and patient stratification was limited to bilateral involvement and familial MD.

Personalized medicine will combine hearing profile and high-resolution magnetic resonance imaging to improve the characterization of the MD phenotype and, together with genomic datasets, will open the door to targeted therapeutic strategies.

**Table 1.** Diagnostic criteria for definite and probable Ménière’s disease (MD) ^1^.

*Definite MD*
A. Two or more spontaneous episodes of vertigo, each lasting 20 minutes to 12 hours
B. Audiometrically documented low- to medium-frequency sensorineural hearing loss in one ear, defining the affected ear on at least one occasion before, during, or after one of the episodes of vertigo
C. Fluctuating aural symptoms (hearing, tinnitus, or fullness) in the affected ear
D. Not better accounted for by another vestibular diagnosis
*Probable MD*
A. Two or more episodes of vertigo or dizziness, each lasting 20 minutes to 24 hours
B. Fluctuating aural symptoms (hearing, tinnitus, or fullness) in the affected ear
C. Not better accounted for by another vestibular diagnosis

Clinical research studies should include only patients with definite MD. Probable MD must be considered when no reliable hearing test has confirmed the temporal relationship between the episode of vertigo and the hearing loss.

## From clinical variants toward personalized Ménière’s disease

Molecular and clinical heterogeneity among patients is very common in multifactorial inflammatory disorders. The immune response to the same antigenic exposure or to immune treatments commonly shows wide inter-individual variations. The transition from symptom-based to molecular-based personalized treatments first requires a deep knowledge of the genetic epidemiology of MD. Therefore, large-scale analyses of the genomic and molecular datasets of individuals experiencing defined disease conditions are required to identify reliable patient-specific biomarkers linking genotypes and single-cell gene expression profiles with endophenotypes. MD could involve one ear (unilateral) or both ears (bilateral MD). There is little clinical evidence supporting the possibility that they have different mechanisms ^22^. In patients with unilateral MD, hearing loss usually involves low and middle frequencies more than high frequencies, even in patients with a long follow-up (>20 years); on the other hand, patients with bilateral MD have an involvement of all frequencies in the audiogram ^22^. So the cochlear damage will be limited to the apical and middle turn in individuals with MD limited to a single ear, but it will affect the entire cochlea in patients with bilateral involvement. However, the major finding of clinical research in MD has been the identification of different clinical subgroups of patients with potentially different etiological factors.

The Ménière’s Disease Consortium (a European multicenter initiative to collect clinical and biological data) has identified five clinical variants among patients with MD ( Table 2). So hierarchical cluster analyses using few categorical variables as predictors can identify subgroups of patients in clinical practice ^20, 21^. Initially, 398 patients with bilateral MD were investigated since they showed a lower variation in the phenotype according to hearing profile. Group or MD type 1, which included 46% of patients, was defined by SNHL starting in one ear and involving the second ear in the following months or years but without migraine and autoimmune comorbidities. Group 2, 17% of patients, was characterized by simultaneous onset of hearing loss in both ears without migraine or autoimmunity. MD type 3 (13% of patients) clustered in families with MD, and group 4 (12% of patients) was associated with migraine in all cases. MD type 5 represented 11% of patients with MD and additionally autoimmune disease.

**Table 2.** Clinical subgroups of patients with unilateral and bilateral Ménière’s disease.

*Unilateral Ménière’s disease (MD)*	
Type 1	Sporadic MD (if concurrent migraine, autoimmune disease, or familial MD is observed, patients are out of this subgroup)
Type 2	Delayed MD (hearing loss precedes vertigo attacks in months or years)
Type 3	Familial MD (at least two patients in the first or second degree)
Type 4	Sporadic MD with migraine (temporal relationship not required)
Type 5	Sporadic MD plus an autoimmune disease
*Bilateral MD*	
Type 1	Unilateral hearing loss becomes bilateral
Type 2	Sporadic, simultaneous hearing loss (usually symmetric)
Type 3	Familial MD (most families have bilateral hearing loss, but unilateral and bilateral cases may coexist in the same family)
Type 4	Sporadic MD with migraine
Type 5	Sporadic MD with an autoimmune disease

The temporal course of the hearing profile distinguishes unilateral or bilateral involvement ^19– 21^.

In a subsequent study, Frejo *et al*. analyzed clinical data from 1,073 patients with unilateral MD with at least 5 years of follow-up since disease onset ^21^. Of note, some of the predictors described in patients with bilateral MD were also confirmed in patients with unilateral involvement. In unilateral MD, group 1 was the clinical variant most frequently observed (53%), and it included patients without a familial history of MD, migraine, or autoimmune comorbidity; MD type 2 was termed delayed MD and was a rare condition (8%) characterized by SNHL which antedated the vertigo episodes; familial MD or type 3 (13%) included all familial cases of MD, although some patients in these families may show unilateral SNHL; MD type 4 (15%) was associated with migraine with or without aura; and MD type 5 (11%) was defined by a concurrent autoimmune disorder.

However, the cluster analysis using the aggregated data of the unilateral and bilateral patients was not able to reproduce the same groups. This is probably due to an increase of heterogeneity pointing to unilateral and bilateral MD being different disorders.

## Evidence for a genetic contribution to Ménière’s disease

Several lines of epidemiological evidence support a genetic contribution in MD, including (a) the higher prevalence observed in the European population over other ethnicities ^16^ and (b) a strong familial aggregation found in Europeans and South Koreans ranging from 6% to 10% of cases with a high sibling recurrence risk ratio **(**λs = 24–45) ^18, 19, 23– 25^.

The genes that have been related to the initiation and progression of MD can be classified into four main categories: (a) cell surface channels, (b) extracellular matrix proteins, (c) immune-associated, and (d) proliferation and cell survival genes.

Small case control studies suggested an association with HLA class II genes in different populations ^26, 27^, but these findings were not further replicated ^28^. However, two genes of the immune response have been associated with hearing loss progression in MD in larger cohorts: *MICA* and *TLR10*. So the carriers of the allelic variant MICA*A.4 showed a slower hearing loss progression, but the significance of this finding remains unknown ^29^. In a separate study, the common variant rs11096955 in the *TLR10* gene also influenced the progression of SNHL in patients with bilateral MD ^30^. The *MIF* gene, encoding macrophage migration inhibitory factor, is a multifunctional cytokine which mediates the production of pro-inflammatory cytokines and enhances autoimmune-mediated neuroinflammation ^31^. Moreover, MIF seems to increase blood–brain barrier permeability ^32^ and may have a role in macrophage activation in MD ^33, 34^. Altogether, these findings support a role for the innate immune response in patients with MD.

More recently, Li *et al*. used a molecular network-based method using a random walk with restart algorithm to predict genes potentially involved in MD ^35^. A total of 43 genes were prioritized, and 11 of them were genes involved with immune-associated biological processes, such as *CD4*, *IL6*, *IL-1R1*, and *TLR2* ^35^. A genome association study using a high-density genotyping array containing 196,524 single-nucleotide variants identified two common allelic variants in the *NFKB1* gene (rs3774937 and rs4648011) that seem to modify the hearing progression in patients with MD ^36^. Nuclear factor-kappa B (NF-κB) is a complex of transcription factors, which regulate the inflammatory response, and its activation by cytokine receptors in lymphoid cells seems to exacerbate the inflammatory response in MD ^37^.

Additional evidence for a genetic contribution is also supported by the exome sequencing studies conducted in four families with MD and an autoimmune background. These rare families showed an autosomal dominant pattern of inheritance and segregated rare variants with potential pathogenic effects in the *FAM136A*, *DTNA*, *PRKCB*, and *DPT* genes ^38– 40^. Although these genes for familial MD should be confirmed in sporadic cases and more families with MD are needed to accept them as causal genes in MD, these findings point to a genetic heterogeneity in familial cases.

Allelic heterogeneity has been found in Korean families with mutations in the *COCH* gene (DFNA9) as well. So, according to the location of the mutation on the gene, distinct vestibular phenotypes have been described; these range from progressive bilateral vestibular loss without episodic vertigo (p.G38D variant) to an MD-like phenotype with severe episodes of vertigo (p.C162Y variant) ^41^.

## Evidence for an autoimmune variant of Ménière’s disease

Different studies have estimated that a third of patients with MD cases may have an immune dysfunction, but the mechanisms involved have not been established ^42^. Several hypotheses have been proposed to explain the development of autoimmune responses in the inner ear: (a) a cross-reaction due to shared epitopes between inner ear proteins and microbial proteins, such as cochlin; (b) collateral damage, as persistent levels of cytokines and chemokines may provoke delayed immune reactions, possibly explaining the relapsing/remitting course of MD; (c) self-intolerance to inner ear antigens; and (d) immunogenetic factors leading to a persistent inflammatory response ^42^.

Autoimmune EH has been induced experimentally by injection of antigens or monoclonal antibodies in murine models ^43^. Several inner ear proteins with molecular weights of 28, 42, 58, and 68 kDa could be the main components inducing autoimmune MD in the guinea pig model ^44^. Additionally, antibodies against type II collagen have been found in the serum of patients with MD ^44^.

Riente *et al*. observed the presence of autoantibodies to inner ear antigens in the serum of patients with MD, but the authors were not able to determine whether the presence of the antibodies occurs before the beginning of symptoms of MD or it is the result of the inner ear inflammation and tissue destruction ^45^.

The cytokine tumor necrosis factor (TNF)-like weak inducer of apoptosis (TWEAK), a member of the TNF superfamily, and the TWEAK/Fn14 pathway have been involved in several autoimmune diseases, including multiple sclerosis, systemic lupus erythematosus, rheumatoid arthritis, or ulcerative colitis ^46^. However, this pathway has only started to be investigated in MD. So the allelic variant rs4947296 is associated with bilateral MD in the Spanish population and has been found in 18% of patients with a comorbid autoimmune disorder ^37^. This region on chromosome 6 is a trans-expression quantitative trait locus and regulates the expression of multiple genes in the TWEAK/Fn14 pathway in peripheral mononuclear cells, leading to an NF-κB-mediated inflammatory response in MD ^37^.

## Evidence for an auto-inflammatory variant of Ménière’s disease

Auto-inflammatory disorders include a spectrum of rare genetic disorders with an innate immune-mediated inflammatory background ^47^. This condition is usually characterized by high levels of the pro-inflammatory cytokine interleukin-1 beta (IL-1β) and the absence of autoantibodies in the sera of patients. This spectrum ranges from familial Mediterranean fever, the first monogenic auto-inflammatory disease described, associated with mutations in the *MEFV* gene ^48, 49^, or cryopyrin-associated periodic fever syndromes, caused by mutations in the *NLRP3* gene ^50^, to complex polygenic auto-inflammatory diseases such as adult-onset Still’s disease, Crohn’s disease, ulcerative colitis, or sarcoidosis ^47^. Several mechanisms have been demonstrated in auto-inflammatory diseases, including (a) IL-1β-mediated auto-inflammation which may involve the NLRP3 inflammasome and its responses to IL-1β blockade ^51^, (b) IL-18-mediated auto-inflammation with mutations in the *NLRC4* gene and macrophage activation syndrome ^52^, and (c) A20 haploinsufficiency with mutations in the *TNFAIP3* gene associated with an increase of pro-inflammatory cytokines such as IL-1β, TNFα, IL-6, IL-18, and IL-17 and NF-κB-mediated inflammation ^53^. Although the evidence to support an auto-inflammatory process is still limited in MD, a case control study has shown that pro-inflammatory cytokines such as IL-1β, IL-1RA, TNFα, and IL-6 were elevated in 21% of MD patients in the supernatant from patient-derived peripheral blood mononuclear cells ^54^. Since these patients did not show any comorbid autoimmune condition, they could be considered within the spectrum of auto-inflammatory disorders. Moreover, extracts from Aspergillus and Penicillium molds triggered a release of TNFα in these patients with high basal levels, which was not observed in the control experiments, suggesting that these molds may contribute to the exacerbation of innate-mediated inflammation in MD ^54^.

## Genetic diagnosis of Ménière’s disease

**Figure 1.**
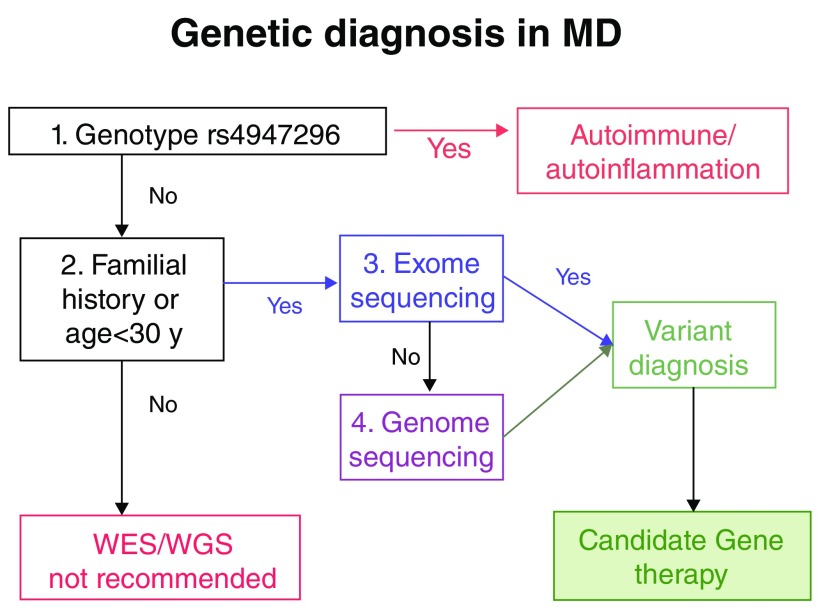
Potential algorithm for the genetic diagnosis of Ménière’s disease (MD). WES, whole exome sequencing; WGS, whole genome sequencing.

Figure 1 lays out a proposal for an algorithm for the genetic diagnosis of MD. Initially, all individuals with a MD phenotype should be tested for rs4947296 to assess whether they have an NF-κB-mediated inflammatory response that is potentially treatable. If a given patient does not carry the risk genotype but presents a familial history of MD or the onset of the disease occurred before the age of 30 years, we recommend performing exome sequencing. Although there are few exome datasets of patients with MD, initially the genetic diagnosis is expected to be possible in 20% to 30% of cases. This diagnosis rate will probably improve with whole genome sequencing, as has occurred with SNHL.

However, if there is no familial history or the onset of the condition occurs over the age of 30 years, we consider the yield of exome or genome sequencing to be very low. Several initiatives such as Genome England ( www.genomicsengland.co.uk) or the Ménière’s Disease Consortium have designed and tested different gene panels for familial and sporadic MD, and the algorithm for the genetic diagnosis of MD will include some of these genes in the near future.

## Conclusions

1. MD should be considered a set of different rare disorders with a core phenotype: episodic vertigo, SNHL, and tinnitus
2. Familial clustering and autosomal dominant MD show a genetic heterogeneity involving several genes such as *COCH*, *DTNA*, *FAM136A, PRKCB*, *SEMA3D*, and *DPT*, but the diagnostic value of genetic testing in patients with sporadic MD remains to be established
3. A personalized treatment should consider the investigation of pro-inflammatory cytokines and rs4947296 as markers of NF-κB-mediated inflammation
4. Genetic diagnosis of MD will pave the way for personalized medicine in vestibular disorders
